# Genetic variants associated with Alzheimer's disease and telomere length

**DOI:** 10.1002/alz70856_107105

**Published:** 2026-01-11

**Authors:** Zainab Khurshid, Tong Tong, Oluwatosin A Olayinka, Congcong Zhu, John J. Farrell, Alzheimer's Disease Sequencing Project (ADSP), Eden R. Martin, William S Bush, Margaret Pericak‐Vance, Li‐San Wang, Gerald D. Schellenberg, Jonathan L Haines, Kathryn L. Lunetta, Xiaoling Zhang, Lindsay A. Farrer

**Affiliations:** ^1^ Department of Medicine (Biomedical Genetics), Boston University Chobanian & Avedisian School of Medicine, Boston, MA, USA; ^2^ Boston University Bioinformatics Program, Boston, MA, USA; ^3^ –, –, PA, USA; ^4^ John P. Hussman Institute for Human Genomics, University of Miami Miller School of Medicine, Miami, FL, USA; ^5^ Department of Population and Quantitative Health Sciences, Case Western Reserve University School of Medicine, Cleveland, OH, USA; ^6^ Penn Neurodegeneration Genomics Center, Department of Pathology and Laboratory Medicine, University of Pennsylvania Perelman School of Medicine, Philadelphia, PA, USA; ^7^ Department of Population & Quantitative Health Sciences, School of Medicine, Case Western Reserve University, Cleveland, OH, USA; ^8^ Department of Biostatistics, Boston University School of Public Health, Boston, MA, USA; ^9^ Biomedical Genetics, Department of Medicine, Boston University Medical School, Boston, MA, USA; ^10^ Bioinformatics Program, Boston University, Boston, MA, USA; ^11^ Department of Epidemiology, Boston University School of Public Health, Boston, MA, USA; ^12^ Department of Neurology and Ophthalmology, Boston University Chobanian & Avedisian School of Medicine, Boston, MA, USA

## Abstract

**Background:**

Telomere length (TL), a marker of biological aging, has been implicated in multiple age‐related diseases, but its association with Alzheimer's disease (AD) remains unclear. We analyzed whole genome sequence data from the Alzheimer's Disease Sequencing Project (release 4) to identify genetic variants associated with both AD and TL.

**Method:**

We estimated TL for 35,014 participants using TelSeq, which after quality control yielded a dataset including 6,973 persons of European ancestry (EA; 3,701 AD cases, 3,273 controls), 4,188 African Americans (AA; 1,329 AD cases, 2,859 controls), 4,008 Caribbean Hispanics (CH; 1,588 AD cases, 2,420 controls), and 4,170 Native American Hispanics (NAH; 754 AD cases, 3,416 controls). The association of AD with TL was tested using a linear regression model including covariates for age, sex, and dosage of the *APOE* e2 and e4 alleles. The interaction of TL with SNPs having a minor allele count >20 was evaluated for its association with AD in each ancestry group by logistic regression models using GENESIS including SNP, TL, an interaction term for SNPxTL, and covariates for age, sex, sequencing center, genetic relationship matrix, and principal components of ancestry. Results were combined across groups using METASOFT.

**Result:**

AD (*p* = 5.32x10^‐12^), age (*p* = 2.00x10^‐16^), sex (*p* = 2.00x10^‐16^), and *APOE* e2 (*p* = 5.35x10^‐6^) were significantly associated with TL. In the EA sample, interaction of TL with *CRTC3‐AS1* intronic SNP rs533691286 was associated with AD at the genome‐wide significance (GWS) level (*p* = 1.13x10^‐8^). Suggestive associations were found with rs537146885 located between *RP5‐884C9.2* and *LINC01343* (*p* = 8.95x10^‐8^). In the NAH group, GWS associations were observed for interactions of TL with *MIR548XHG* intronic variants rs117856971 (*p* = 2.06x10^‐9^) and rs18578882 (*p* = 3.70x10^‐8^). No TLxSNP interactions were GWS in the CH and AA groups. In the total sample, GWS associations were identified for interactions of TL with intronic variants in *SLC23A2 (*rs184956772, *p* = 3.19x10^‐9^) and *CFAP61* (rs769676169, *p* = 3.64x10^‐9^), rs1037982496 upstream of *DEFB123* (*p* = 2.96x10^‐9^), and several intergenic variants including *DEFB115‐DKKL1P1* (rs968020553, *p* = 2.96X10^‐9^), *RP5‐884C9.2‐LINC01343* (rs537146885, *p* = 4.98x10^‐9^), *MIR3193‐COX4I2* (rs921951239, *p* = 2.70x10^‐9^), *AC010091.1* (rs552422184, *p* = 5.86x10^‐9^), and *CTA‐109P11.1‐RP11‐328K15.1 (*rs77699417, *p* = 6.51x10^‐9^).

**Conclusion:**

We identified variants that significantly impact AD risk through their interaction with TL, suggesting that TL maintenance pathways may be central to AD pathogenesis.

